# Dementia Fear Profiles and Influencing Factors Among Older Adults With Multimorbidity in Long‐Term Care Facilities

**DOI:** 10.1002/nop2.71023

**Published:** 2026-11-07

**Authors:** Guiyun Wang, Yuehua Han, Qirong Chen, Yanxia Qi, Jinke Kuang, Chaowei Guo, Shuxing Li

**Affiliations:** ^1^ School of Nursing Shandong Xiehe University Jinan Shandong China; ^2^ Xiangya School of Nursing Central South University Changsha Hunan China; ^3^ Xiangya Center for Evidence‐Based Practice & Healthcare Innovation: A Joanna Briggs Institute Affiliated Group Changsha Hunan China; ^4^ School of Nursing and Rehabilitation North China University of Science and Technology Tangshan Hebei China

**Keywords:** dementia fear, latent profile analysis, long‐term care (LTC) facilities, older adults with multimorbidity

## Abstract

**Aims:**

To identify distinct dementia fear profiles among older adults with multimorbidity in long‐term care facilities and to explore the variables associated with these profiles.

**Design:**

A cross‐sectional study.

**Methods:**

This study included 557 older adults with multimorbidity across 25 LTC facilities. Dementia fear, dementia knowledge, psychological resilience, healthy lifestyle and activities of daily living (ADLs) were assessed with validated tools. Data were collected from March to November 2023. Latent profile analysis (LPA) was used to identify dementia fear profiles, and multinomial logistic regression was applied to explore their associations with relevant variables.

**Results:**

Four latent profiles were identified: low dementia fear—little concerns about family burden (13.3%), low dementia fear—moderate concerns about family burden (33.2%), moderate dementia fear—high concerns about family burden (40.0%), and high dementia fear—moderate concerns about family burden (13.5%). Family history of dementia, co‐residing with a spouse/partner in LTC facilities, psychological resilience, healthy lifestyle and ADLs were significantly linked to membership in the different dementia fear profiles (*p* < 0.05).

**Conclusion:**

Dementia fear among older adults with multimorbidity in long‐term care facilities demonstrates significant heterogeneity. These findings provide a basis for identifying high‐risk subgroups and informing the development of profile‐specific nursing interventions.

**Implications for the Profession and/or Patient Care:**

Nurses in LTC facilities can routinely screen for fear of dementia in older adults with multimorbidity, covering both overall fear levels and specific dimensions such as family burden concerns, and deliver profile‐tailored care. Priority should be given to the largest subgroup of residents with moderate dementia fear and high family burden concerns, with a focus on alleviating family burden, promoting healthy lifestyles and supporting functional independence.

**Reporting Method:**

This study followed the STROBE statement of cross‐sectional studies.

**Patient or Public Contribution:**

No patient or public contribution.

## 1 Introduction

Driven by socioeconomic progress and rapid population aging, nearly universal gains in life expectancy have coincided with an expanded pool of individuals with chronic diseases and a consequent surge in global multimorbidity rates (Palladino et al. 2019). A comprehensive systematic review of 126 population‐based studies (spanning 54 countries) revealed that over half of older adults aged ≥ 60 years live with multimorbidity, highlighting its status as a global public health imperative (Chowdhury et al. 2023). Individuals with multimorbidity bear significant physical and psychological burdens due to high healthcare costs, prolonged disease duration, frequent relapse and increased mortality risk (Foo et al. 2020).

Critically, multimorbidity is not merely a comorbidity cluster but a synergistic driver of neuropathology (Valletta et al. 2024). Chronic conditions converge on pathways of vascular injury, neuroinflammation, and metabolic dysregulation, core mechanisms underpinning neurodegenerative cascades in dementia (Ren et al. 2024). In one prospective cohort study, multimorbidity was associated with a 2.36‐fold increased risk of incident dementia (Ben Hassen et al. 2022). Additionally, this elevated dementia incidence triggers a bidirectional cascade: cognitive impairment further disrupts the management of pre‐existing comorbidities, culminating in accelerated health deterioration and overwhelming care demands (Jia et al. 2020). This heightened dementia risk and the accompanying psychological burden pose a pressing challenge for nursing professionals in long‐term care (LTC) settings, highlighting the need to integrate dementia fear assessment into routine care for residents living with multimorbidity.

## 2 Background

In 2012, Kessler et al. (2012) introduced the concept of dementia fear, defining it as an individual's emotional response to the perceived threat of dementia. Ranked as the second most dreaded condition after cancer (Watson et al. 2023), dementia elicits profound apprehension. Elevated dementia fear not only heightens dementia vulnerability but also compromises daily functioning. In clinically severe presentations, it manifests as significant depression and increased suicidality risk (Cui et al. 2020). Research indicates that the intensification of dementia fear correlates with multiple determinants, including the female sex (Chen et al. 2026), lower educational attainment (Sun et al. 2023), subjective memory complaints or perceived cognitive decline (Chen et al. 2026), suboptimal physical health (Meyer et al. 2024), lower psychological resilience (Dai et al. 2023) and inadequate dementia literacy (Wang et al. 2026). Furthermore, older adults residing in LTC facilities inherently face unique vulnerabilities due to their institutionalised environment and insufficient family care support (Chen et al. 2023). Among these residents, those with multimorbidity experience further amplified vulnerability due to the complex demands of managing multiple chronic conditions. On the one hand, their frequent exposure to residents with dementia within LTC settings directly elicits dementia fear (Han et al. 2023). On the other hand, compared to adults without multimorbidity, those with multimorbidity exhibit more subjective and pronounced fear of this irreversible disease, as they have personally experienced the health detriments caused by their multiple chronic conditions (Du et al. 2024). This dual effect results in substantially higher levels of concern about developing dementia among these individuals compared to community‐dwelling older adults.

Recognising and responding to these concerns falls largely to frontline nurses in LTC settings. Within LTC facilities, nurses have the most sustained contact with residents and are responsible for admission assessment, routine observation, health education and care planning (Han and Shao 2026). Nurses are therefore uniquely positioned to identify and address residents' emotional responses to dementia risk. Routine assessment in LTC settings, however, typically covers physical function, nutrition, falls risk and cognitive screening, and emotional concerns about dementia are rarely assessed in a structured way (Werner et al. 2021). Unrecognised dementia fear can affect nursing care in observable ways, including avoidance of cognitive screening and help‐seeking, reduced engagement in health‐promoting and rehabilitative activities, and escalating care needs (Orom and Allard 2025). This highlights the need for a clearer understanding of heterogeneity in dementia fear that can inform how nurses identify and respond to differing psychological needs in routine LTC practice.

Current research on dementia fear primarily focuses on middle‐aged individuals or community‐dwelling older adults (Maxfield et al. 2024; Meyer et al. 2024). However, this topic remains significantly understudied in LTC facilities, where the care environment, social interactions and health needs of institutionalised older adults differ fundamentally from those of community‐dwelling populations, making it difficult to directly generalise existing findings to this setting. Research is even scarcer among the high‐risk subgroup of institutionalised older adults with multimorbidity. Methodologically, existing studies predominantly rely on total scores of dementia fear scales to represent overall severity (Lee and Jung 2020; Meyer et al. 2024), neglecting the distinct characteristics of its dimensions and the inherent heterogeneity within populations. This variable‐centred, unidimensional approach may obscure clinically meaningful subgroups with distinct combinations of dementia fear and family burden concerns, thereby limiting nurses' ability to recognise differing psychological needs and deliver targeted, person‐centred support in LTC settings. Latent profile analysis (LPA), a person‐centred analytical approach, has been applied to identify meaningful subgroups in community‐dwelling adult populations (Werner et al. 2026) and hospitalised older adults in China (Guo et al. 2025), revealing that dementia fear comprises qualitatively distinct configurations. Both studies sampled community or hospital populations rather than LTC residents. This distinction is particularly relevant for nursing practice, as hospital care is short‐term and focused on acute treatment, whereas LTC facilities provide long‐term residential care settings (Harrison et al. 2025). To our knowledge, no study has applied LPA to identify dementia fear profiles among institutionalised older adults with multimorbidity. Without such evidence, nurses have no empirical basis for identifying which residents may be experiencing different patterns of dementia fear, or what specific types of concerns to focus on when assessing and supporting their psychological well‐being.

This study was conducted in comprehensive LTC facilities that provide services for older adults with normal cognitive function as well as those with varying levels of cognitive impairment. We focused on residents with intact cognitive function but multiple chronic conditions. This population is in a critical window for the primary prevention of dementia (Xin et al. 2025). Dementia fear among these individuals therefore not only represents a major psychological burden but also serves as an important and modifiable clinical target. To address research gaps and limitations in existing literature, this study used LPA with two core research objectives: (1) to identify distinct dementia fear profiles within this vulnerable population with complex health needs, and (2) to explore the factors associated with these different profiles. Ultimately, this work aims to better understand patterns of dementia fear and related psychological distress among older adults under the dual pressures of multimorbidity and institutional LTC. By providing nurses with an empirical basis for recognising differing dementia fear patterns in routine care, the findings have important implications for nursing assessment, staff education and the prioritisation of psychological support under existing staffing constraints.

## 3 The Study

### 3.1 Aims

This study aimed to identify distinct latent profiles of dementia fear among older adults with multimorbidity living in LTC facilities and to examine their associations with sociodemographic, clinical and psychosocial factors.

## 4 Methods

### 4.1 Design

This is a cross‐sectional study. The study was reported in accordance with the Strengthening the Reporting of Observational Studies in Epidemiology (STROBE) guidelines (Vandenbroucke et al. 2007).

### 4.2 Instrument With Validity and Reliability

#### 4.2.1 Demographic Characteristics Questionnaire

The research team reviewed relevant literature and designed an information questionnaire including age, sex, educational background, occupation, living burden (defined as participants' self‐reported subjective current economic burden, coded as a 3‐level ordinal variable), family history of dementia, co‐residing with a spouse/partner in LTC, residence, number of children and duration of stay in a nursing home.

#### 4.2.2 Fear of Dementia Scale (FODS)

The Fear of Dementia Scale was developed by Lee and Jung (2020) to measure dementia fear and has been translated and validated among Chinese populations (Xu et al. 2021). The survey includes experience and cognition of dementia (7 items), concerns about family burden (4 items), shame (3 items) and physical response (4 items), totalling 18 items. Participants rated each item on a five‐point Likert scale ranging from 1 (strongly disagree) to 5 (strongly agree). The initial score ranges from 18 to 90, with higher scores indicating a stronger dementia fear. FODS has good reliability and validity. In this study, the Cronbach's *α* coefficient for the total scale was 0.923, and the coefficients for the four subscales were 0.873, 0.910, 0.820 and 0.965, respectively.

#### 4.2.3 Dementia Knowledge Assessment Scale (DKAS)

The Dementia Knowledge Assessment Scale was developed by Annear et al. to measure dementia knowledge (Annear et al. 2015). The Chinese version was translated and validated by Zhang et al. (2019). This questionnaire includes 25 items in four dimensions: causes and characteristics (7 items), communication and engagement (6 items), care needs (6 items) and risks and health promotion (6 items). Using the 0–2 grade scoring method (2 = correct, 1 = probably correct, 0 = wrong, probably wrong or don't know), the total score ranged from 0 to 50, with higher scores representing greater dementia knowledge. The DKAS has good reliability and validity. The Cronbach's *α* coefficient in this study was 0.845.

#### 4.2.4 Connor–Davidson Resilience Scale (CD‐RISC)

The Connor–Davidson Resilience Scale was developed to measure psychological resilience (Connor and Davidson 2003) and was revised and validated by Chinese researchers (Yu et al. 2007). It includes three dimensions: tenacity (13 items), strength (8 items) and optimism (4 items), totalling 25 items. Participants rated each item on a five‐point Likert scale from 0 (never) to 4 (all the time). The initial score ranges from 0 to 100, with higher scores meaning greater psychological resilience. The Cronbach's *α* coefficient in this study was 0.934.

#### 4.2.5 Health‐Promoting Lifestyle Profile II (HPLP‐IIR)

The Health‐Promoting Lifestyle Profile II was developed and presented by Cao et al. (2016) to measure healthy lifestyle. It includes six dimensions: physical activity (8 items), health responsibility (11 items), stress management (5 items), nutrition (6 items), interpersonal relations (5 items) and spiritual growth (5 items), with a total of 40 items. Participants rated each item on a four‐point Likert scale from 1 (never) to 4 (all the time). The initial score ranges from 40 to 160, with higher scores indicating healthier lifestyles. The Cronbach's *α* coefficient in this study was 0.925. It should be noted that some subscales of the HPLP‐IIR had relatively low alpha coefficients in the original validation study (as low as 0.63) (Cao et al. 2016), below the conventional threshold of 0.70. Therefore, the total score was used in the primary analyses.

#### 4.2.6 The Barthel Index (BI)

The Barthel Index was developed by Mahoney and Barthel (1965) and has also been translated and validated by Chinese scholars (Cai et al. 2007). It is used to measure ADLs. There are two versions: one with 10 items, and one with 15 items. The 10‐item version includes feeding, bathing, grooming, dressing, bowels, bladder, using the toilet, Transfer: Chair/bed, walking on flat ground and climbing stairs. The total score ranged from 0 to 100, with the final scores categorised as follows: severe dysfunction (0–40), moderate dysfunction (41–60), mild dysfunction (61–99) and complete self‐care (100). The Cronbach's *α* coefficient in this study was 0.855.

### 4.3 Sampling and Recruitment

A random cluster sampling method was employed to recruit older adults with multimorbidity residing in LTC facilities in Tangshan, China. All registered and operational LTC facilities in Tangshan (*n* = 360) were identified through the Tangshan Municipal Civil Affairs Bureau. These facilities were uniformly numbered from 001 to 360. Using a random number table, the facilities were sequentially selected one by one until the predetermined sample size was reached.

### 4.4 Sample Size and Power

Current guidelines recommend a minimum sample size of > 500 for robust LPA (Sinha et al. 2021). Additionally, in cross‐sectional studies, the sample size should typically be 10–20 times the number of observed variables (Ni et al. 2010). With 27 variables included in this study and accounting for an estimated 20% rate of invalid responses, the calculated required sample size ranged from 338 to 675. A total of 570 questionnaires were distributed; 560 were returned (98.2% return rate). Of the 10 non‐returned questionnaires, reasons included interruption of the completion session by other commitments (e.g., scheduled activities, treatment or family visits) and fatigue during questionnaire completion. Three returned questionnaires were excluded as invalid because more than 20% of items were unanswered or the responses were logically inconsistent, yielding 557 valid questionnaires (effective response rate 99.5%). Among the 557 valid questionnaires, item completeness was checked by researchers on site at the time of collection; there were no item‐level missing data, and no imputation was performed.

### 4.5 Population and Sample

The target population comprised older adults with multimorbidity residing in LTC facilities. The study sample included 557 older adults with multimorbidity from 25 facilities across 10 districts/counties.

### 4.6 Inclusion and Exclusion Criteria

The inclusion criteria were as follows: (1) age ≥ 60 years; (2) a diagnosis of ≥ 2 chronic diseases; and (3) residence in the facility for ≥ 3 months.

The exclusion criteria were as follows: (1) cognitive impairment, defined by trained study investigators during on‐site facility visits as a Mini‐Mental State Examination (MMSE) score of < 24 (Creavin et al. 2016); (2) severe visual or auditory impairment that could not be adequately corrected to allow participation; and (3) communication impairment that precluded valid questionnaire completion.

### 4.7 Data Collection

The data were collected between March 2023 and November 2023. On‐site paper questionnaires were administered to eligible older adults with multimorbidity in LTC facilities. Prior to distribution, the researcher explained the study's purpose, procedures and completion methods, obtaining written informed consent and assuring patients of data confidentiality and exclusive academic use. Given the low educational level of the study population, the questionnaire was commonly administered via researcher‐assisted face‐to‐face interviews. Trained researchers read each item aloud using standardised wording and recorded participants' responses sequentially without interpretation or prompting. Participants with sufficient literacy could complete the questionnaire under researcher supervision, with clarification available when needed. To minimise the potential interviewer effect of oral administration, all researchers completed unified standardised training on survey administration and standardisation practice before the study. A fixed scripted wording manual was used for all oral administration procedures, and full‐process quality control was implemented through pre‐survey consistency checks, double data entry review and on‐site supervision to ensure the reliability and homogeneity of the collected data.

### 4.8 Data Analysis

Data were analysed using Mplus version 8.3, IBM SPSS Statistics version 26.0, and R. Categorical variables were summarised using frequencies and percentages, while continuous variables were summarised as means (SD). Bivariate associations among dementia fear, dementia knowledge, psychological resilience, health‐promoting lifestyle and activities of daily living (ADLs) were examined using Pearson's correlation coefficients (*r*). The correlation matrix was visualised as a heatmap using R.

LPA was used as the principal person‐centred analytical approach to identify unobserved subgroups characterised by distinct patterns across the four dimensions of dementia fear. Unlike variable‐centred methods, which primarily estimate associations among variables at the overall sample level, LPA allows individuals to be grouped according to similarities in their response patterns without assuming that the study population is homogeneous (Bergman and Magnusson 1997; Ferguson et al. 2019). The four FODS subscale scores were used as profile indicators. To place the indicators on a common metric, the raw subscale scores were transformed into T‐scores using the formula *T* = 50 + 10[(*X* − *M*)/SD], where *X* represents the raw score, *M* the sample mean and SD the standard deviation. The resulting indicators had a mean of 50 and a standard deviation of 10 (Ferguson et al. 2019; Spurk et al. 2020).

Models containing one to five profiles were estimated sequentially. Model selection was based on a combination of statistical fit, classification quality, parsimony, profile size and substantive interpretability. Lower values of the Akaike information criterion (AIC), Bayesian information criterion (BIC) and sample‐size‐adjusted Bayesian information criterion (aBIC) indicated better relative model fit. Changes in these information criteria across successive models were also examined using two‐dimensional line plots to identify diminishing improvements in fit (Petras and Masyn 2010). Entropy values closer to 1 indicated greater classification accuracy, with values > 0.80 considered acceptable (Wong et al. 2018). The Lo–Mendell–Rubin likelihood ratio test (LMR) and bootstrap likelihood ratio test (BLRT) were used to compare each *k*‐profile model with the model containing one fewer profile. A statistically significant result indicated that the *k*‐profile solution provided a better fit than the (*k* − 1)‐profile solution (Kim 2014). Profiles representing < 5% of the sample were considered potentially unstable and were taken into account when evaluating model adequacy (Ferguson et al. 2019).

Between‐profile differences in categorical variables were examined using Pearson's chi‐squared test or Fisher's exact test, as appropriate. Differences in continuous variables, including DKAS, CD‐RISC, HPLP‐IIR and BI scores, were assessed using Welch's analysis of variance because the homogeneity‐of‐variance assumption was not met. Tamhane's *T*2 test was used for post hoc pairwise comparisons. Within each row, profile means marked with different superscript letters (a, b and c) differed significantly, whereas means sharing the same superscript letter did not differ significantly.

Because profile membership was a nominal outcome with four categories, multinomial logistic regression was used to examine factors associated with profile membership, with Profile 1 serving as the reference category. Adjusted odds ratios (ORs), 95% confidence intervals (CIs), and *p*‐values were reported. Multicollinearity among independent variables was assessed using variance inflation factors (VIF), with all values below 2.0 indicating no concerning collinearity. LPA was conducted using Mplus version 8.3. Descriptive, bivariate, between‐profile and regression analyses were conducted using IBM SPSS Statistics version 26.0, and the correlation heatmap was generated using R. All statistical tests were two‐tailed, and *p* < 0.05 was considered statistically significant.

### 4.9 Ethical Considerations

This study was conducted in accordance with the Declaration of Helsinki. Ethical approval was granted by the Ethics Review Committee of North China University of Science and Technology (approval number: 20211104, approval date: 4 November 2021). Participants were fully informed of the study objectives, the voluntary nature of participation, and the protocols implemented to protect confidentiality and anonymity. All questionnaires were assigned unique numerical identifiers with no personally identifiable information, and no identifiable personal data will appear in any published outputs. Written informed consent was obtained from all participants after they fully understood the purpose and procedures of the study.

## 5 Results

### 5.1 Sociodemographic and Clinical Characteristics of the Participants

A total of 557 participants participated. The majority of the survey respondents were aged ≥ 80 years (*n* = 281, accounting for 50.4% of the sample). A total of 226 were men (40.6%), and 331 were women (59.4%). Their literacy levels were mostly elementary school and below, accounting for 73.2%. Most participants were agricultural labourers (*n* = 262, 47.0%). More demographic information is shown in Table 1.

**TABLE 1:** Demographics and clinical characteristics of the participants (*n* = 557).

Variables	*n* (%)
Age	
60–69	63 (11.3)
70–79	213 (38.2)
≥ 80	281 (50.4)
Sex	
Male	226 (40.6)
Female	331 (59.4)
Education background	
Primary and below	408 (73.2)
Junior high school	92 (16.5)
Senior high school	31 (5.6)
College school and above	26 (4.7)
Occupation	
Not working	104 (18.7)
Agricultural labour	262 (47.0)
Professional personnel	86 (15.4)
Managerial personnel	56 (10.1)
Others	49 (8.8)
Living burden	
No burden at all	330 (59.2)
Basically no burden	202 (36.3)
Have a certain burden	25 (4.5)
Family history of dementia	
No	362 (65.0)
Yes	195 (35.0)
Co‐residing with a spouse/partner in LTC	
No (separated, single, divorced, widowed)	424 (76.1)
Yes	133 (23.9)
Residence	
City	132 (23.7)
Countryside	425 (76.3)
Number of children	
0–1	107 (19.2)
2	160 (28.7)
≥ 3	290 (52.1)
Duration of stay in a nursing home	
≤ 6 months	172 (30.9)
6–12 months	45 (8.1)
≥ 12 months	340 (61.0)

In terms of clinical multimorbidity characteristics, the mean number of chronic conditions per participant was 2.30 (SD = 0.504), with a median of 2 and an interquartile range of 2–3 conditions. The distribution of chronic conditions was as follows: 400 participants (71.8% of the study population) had two chronic conditions, 147 (26.4%) had three, and 10 (1.8%) had four or more. Within this sample, the most common two‐disease multimorbidity cluster was hypertension co‐occurring with diabetes (112 participants, 20.1%), followed by hypertension with stroke (74 participants, 13.3%). The most frequent three‐disease multimorbidity cluster was hypertension, diabetes and coronary heart disease (72 participants, 12.9%).

### 5.2 Bivariate Correlations Among Study Variables and Dementia Fear

Significant inverse correlations were observed between dementia fear and dementia knowledge (*r* = −0.351, *p* < 0.001), psychological resilience (*r* = −0.124, *p* < 0.01), healthy lifestyle (*r* = −0.258, *p* < 0.001) and ADLs (*r* = −0.308, *p* < 0.001), as presented in Figure 1.

**FIGURE 1:**
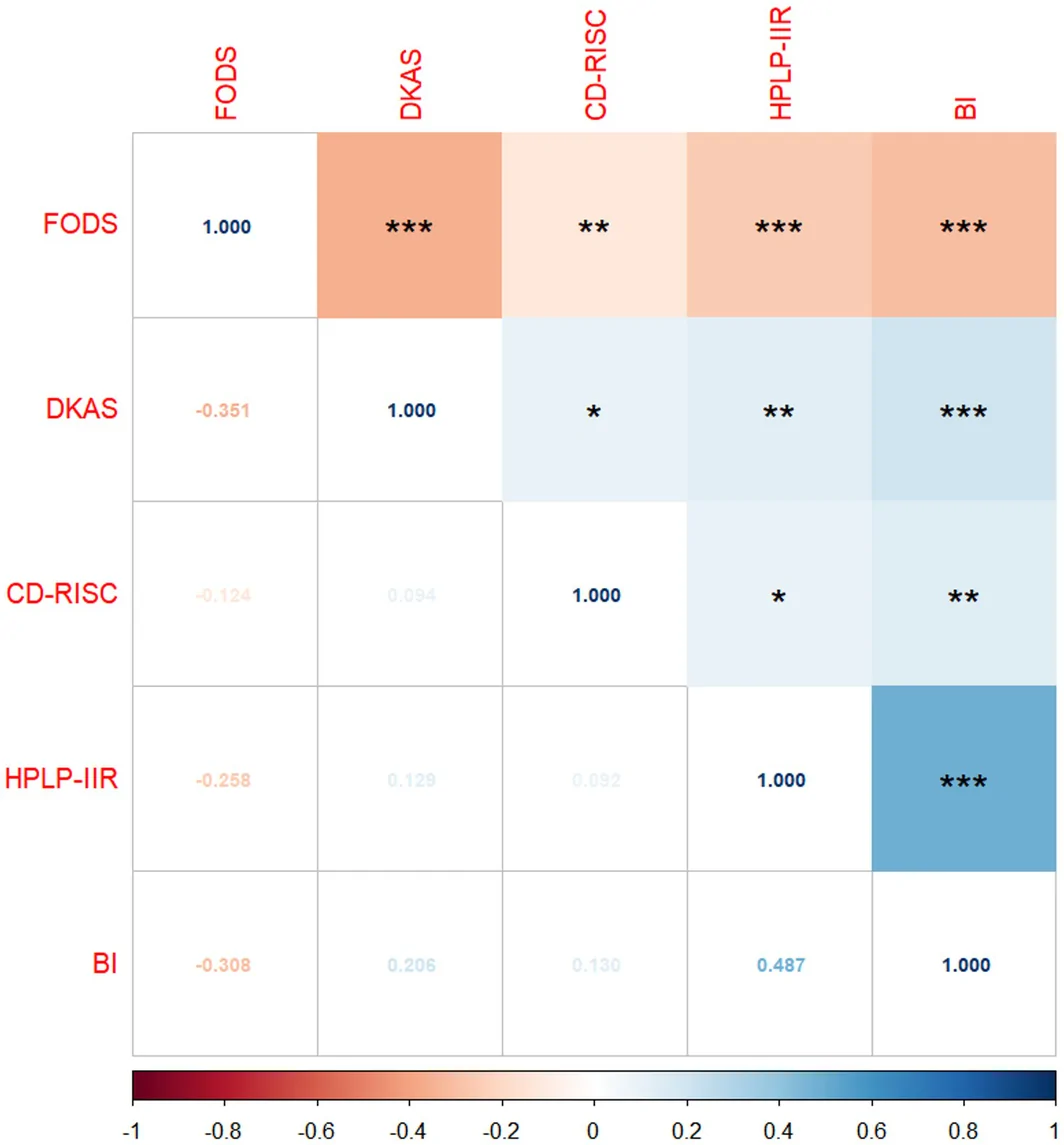
Correlation heatmap among main study variables. Each cell displays pairwise Pearson correlation coefficients. Colour gradient represents correlation values ranging from −1 to 1. BI, The Barthel index; CD‐RISC, Connor–Davidson Resilience Scale; DKAS, Dementia Knowledge Assessment Scale; FODS, Fear of Dementia Scale; HPLP‐IIR, The Health‐Promoting Lifestyle Profile II. ***p < 0.001; **p < 0.01; *p < 0.05.

### 5.3 Latent Profile Analysis and Naming of Dementia Fear

The results of the LPA indicated that a four‐profile model best fit the FODS data among participants. First, entropy values increased sequentially across the two‐ to five‐profile models, ranging from 0.866 to 0.945, with all values exceeding the recommended threshold of 0.8 for good classification accuracy. The LMR and BLRT for the two‐, three‐ and four‐profile models were statistically significant (*p* < 0.05), indicating that each *k*‐profile model demonstrated a significant improvement over the (*k* − 1)‐profile model. However, the LMR test for the five‐profile solution was not significant (*p* = 0.223), and neither was the BLRT (*p* = 0.246), indicating that adding a fifth profile did not significantly improve model fit beyond the four‐profile solution (Table 2).

**TABLE 2:** Comparison of fit indices between models.

Profile	AIC	BIC	aBIC	Entropy	LMR	BLRT	Profile prevalence
					*p*	*p*	
1	16595.106	16629.686	16604.290	—	—	—	—
2	15848.729	15904.922	15863.654	0.866	< 0.001	< 0.001	0.452/0.548
3	15553.215	15631.021	15573.881	0.911	< 0.001	< 0.001	0.118/0.388/0.494
4	15394.003	15493.422	15420.409	0.934	0.012	< 0.001	0.133/0.332/0.400/0.135
5	15299.412	15420.444	15331.559	0.945	0.223	0.246	0.059/0.078/0.397/0.324/0.142

Abbreviations: aBIC, Sample‐Size Adjusted BIC; AIC, Akaike Information Criterion; BIC, Bayesian Information Criterion; BLRT, Bootstrap Likelihood Ratio Test; LMR, Lo–Mendell–Rubin Adjusted Likelihood Ratio Test.

As shown in Figure S1 (Line plot of LPA fit indices for the FODS), the rate of decline in information criteria also slowed noticeably from the four‐ to five‐profile models, supporting an elbow at the four‐profile solution. Combined with the non‐significant LMR/BLRT for the five‐profile model, high classification accuracy (entropy = 0.934) and clinical interpretability of the profile characteristics, the four‐profile solution was retained as the final model. Four distinct FODS profiles were observed among participants, as presented in Figure 2.

**FIGURE 2:**
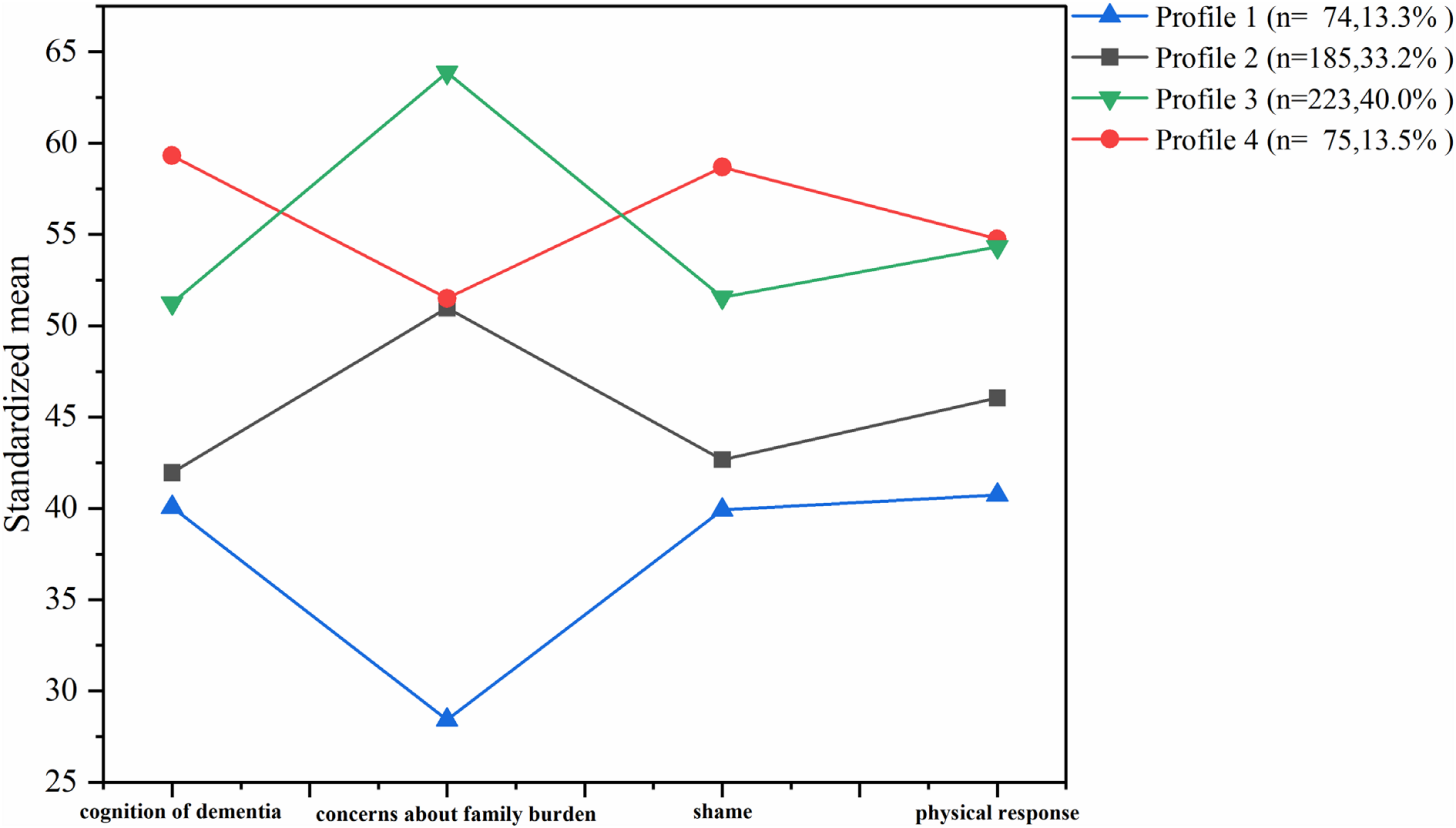
Latent profile of the FODS among the older people with multimorbidity.

The first group, comprising 74 individuals (13.3%), was classified as the ‘low dementia fear—little concerns about family burden’ group. The second group included 185 individuals (33.2%) and was labelled ‘low dementia fear—moderate concerns about family burden’. The third group was the largest, with 223 individuals (40.0%), and was designated ‘moderate dementia fear—high concerns about family burden’. The fourth group comprised 75 individuals (13.5%), displayed the highest level of fear, and was designated ‘high dementia fear—moderate concerns about family burden’.

### 5.4 Factors Associated With Distinct Dementia Fear Profiles

For categorical variables including gender, living burden, family history of dementia, co‐residing with a spouse/partner in LTC, and length of nursing home stay, significant differences were observed across the four dementia fear profiles via chi‐squared tests or Fisher's exact tests. For continuous variables, Welch's ANOVA revealed statistically significant between‐profile differences in scores of the DKAS, CD‐RISC, HPLP‐IIR and BI. Post hoc pairwise comparisons performed with Tamhane's *T*2 further showed that Profile 3 had the lowest DKAS scores: its DKAS scores were significantly lower than those of Profiles 1 and 2 but not Profile 4. In contrast, Profile 3 had the lowest CD‐RISC scores, which were significantly lower than those of all other profiles. Profile 1 had the highest HPLP‐IIR and BI scores, significantly higher than those of all other profiles, whereas Profiles 2 and 3 did not differ significantly on either measure (Table 3).

**TABLE 3:** Differences in demographic and continuous variables among the latent profile (*n* = 557).

Variables	Groups				Test statistic	*p*
	Profile 1 (*n* = 74)	Profile 2 (*n* = 185)	Profile 3 (*n* = 223)	Profile 4 (*n* = 75)		
Age					*χ* ^2^ = 7.520	0.275
60–69	7 (9.5)	27 (14.6)	20 (9.0)	9 (12.0)		
70–79	28 (37.8)	77 (41.6)	78 (35.0)	30 (40.0)		
≥ 80	39 (52.7)	81 (43.8)	125 (56.1)	36 (48.0)		
Sex					*χ* ^2^ = 8.193	0.042
Male	40 (54.1)	70 (37.8)	82 (36.8)	34 (45.3)		
Female	34 (45.9)	115 (62.2)	141 (63.2)	41 (54.7)		
Education background					—	0.324
Primary and below	50 (67.6)	142 (76.8)	169 (75.8)	47 (62.7)		
Junior high school	13 (17.6)	27 (14.6)	32 (14.3)	20 (26.7)		
Senior high school	6 (8.1)	8 (4.3)	12 (5.4)	5 (6.6)		
College school and above	5 (6.8)	8 (4.3)	10 (4.5)	3 (4.0)		
Occupation					*χ* ^2^ = 14.863	0.249
Not working	10 (13.5)	32 (17.3)	44 (19.7)	18 (24.0)		
Agricultural labour	33 (44.6)	92 (49.7)	106 (47.5)	31 (41.3)		
Professional personnel	16 (21.6)	24 (13.0)	36 (16.1)	10 (13.3)		
Managerial personnel	9 (12.2)	17 (9.2)	17 (7.6)	13 (17.3)		
Others	6 (8.1)	20 (10.8)	20 (9.0)	3 (4.0)		
Living burden					*χ* ^2^ = 13.071	0.042
No burden at all	52 (70.3)	104 (56.2)	139 (62.3)	35 (46.7)		
Basically no burden	20 (27.0)	75 (40.5)	71 (31.8)	36 (48.0)		
Have a certain burden	2 (2.7)	6 (3.2)	13 (5.8)	4 (5.3)		
Family history of dementia					—	< 0.001
No	70 (94.6)	181 (97.8)	70 (31.4)	41 (54.7)		
Yes	4 (5.4)	4 (2.2)	153 (68.6)	34 (45.3)		
Co‐residing with a spouse/partner in LTC					*χ* ^2^ = 9.848	0.020
No (separated, single, divorced, widowed)	66 (89.2)	139 (75.1)	168 (75.3)	51 (68.0)		
Yes	8 (10.8)	46 (24.9)	55 (24.7)	24 (32.0)		
Residence					*χ* ^2^ = 6.235	0.101
City	16 (21.6)	49 (26.5)	43 (19.3)	24 (32.0)		
Countryside	58 (78.4)	136 (73.5)	180 (80.7)	51 (68.0)		
Number of children					*χ* ^2^ = 7.805	0.253
0–1	18 (24.3)	29 (15.7)	40 (17.9)	20 (26.7)		
2	18 (24.3)	62 (33.5)	60 (26.9)	20 (26.7)		
≥ 3	38 (51.4)	94 (50.8)	123 (55.2)	35 (46.7)		
Duration of stay in a nursing home					*χ* ^2^ = 16.674	0.011
≤ 6 months	21 (28.4)	55 (29.7)	73 (32.7)	23 (30.7)		
6–12 months	3 (4.1)	27 (14.6)	11 (4.9)	4 (5.3)		
≥ 12 months	50 (67.6)	103 (55.7)	139 (62.3)	48 (64.0)		
DKAS	18.65 (6.787)^ab^	19.06 (6.805)^a^	15.56 (4.879)^c^	16.92 (3.436)^bc^	*F*_w_ = 13.419	< 0.001
CD‐RISC	31.65 (11.777)^a^	32.61 (14.712)^a^	25.93 (11.408)^b^	35.47 (5.006)^a^	*F*_w_ = 32.951	< 0.001
HPLP‐IIR	87.11 (14.825)^a^	75.48 (14.502)^b^	77.99 (11.193)^b^	62.60 (6.142)^c^	*F*_w_ = 108.370	< 0.001
BI	88.51 (14.822)^a^	78.95 (18.440)^b^	76.86 (14.773)^b^	65.00 (21.514)^c^	*F*_w_ = 21.897	< 0.001

*Note:* Categorical variables are presented as *n* (%); continuous variables are presented as mean (SD). ‘—’ indicates Fisher exact probability; Welch's ANOVA was used for continuous variables (due to heterogeneity of variance), with *F*_w_ representing Welch's *F*‐statistic and Tamhane's *T*2 for post hoc tests. Within each row, different superscript letters (a, b, c) denote significant between‐profile differences (*p* < 0.05); shared letters denote no significance. Profile 1 = Low dementia fear—little concerns about family burden, Profile 2 = Low dementia fear—moderate concerns about family burden, Profile 3 = Moderate dementia fear—high concerns about family burden, Profile 4 = High dementia fear—moderate concerns about family burden.

Abbreviations: BI, The Barthel index; CD‐RISC, Connor–Davidson Resilience Scale; DKAS, Dementia Knowledge Assessment Scale; HPLP‐IIR, The Health—Promoting Lifestyle Profile II.

This profile‐level pattern contrasts with the overall significant negative bivariate correlation between dementia fear and psychological resilience. Although the overall trend indicates that greater dementia fear is associated with lower resilience, this relationship is not simply linear across latent profiles: Profile 4, despite having the highest overall dementia fear score, had the highest mean resilience, whereas Profile 3, with only moderate fear, had the lowest. This suggests that the association between resilience and dementia fear is not uniform but varies across profile subgroups.

A multinomial logistic regression analysis was performed to explore the factors linked to the distinct dementia fear profiles in older people with multimorbidity. The analysis incorporated significant variables identified in the preceding univariate analysis as independent predictors, while dementia fear profiles were considered the outcome variable.

1. Profile 1 (Low dementia fear—little concerns about family burden): This profile was used as the reference category. For all categorical variables, reference categories were labelled with superscript ‘a’ in Table 4, including Sex (Female), Living burden (No burden at all), Co‐residing with a spouse/partner in LTC (Yes), etc. Multinomial regression identified the factors associated with dementia fear profiles.
2. Profile 2 (Low dementia fear—moderate concerns about family burden): The absence of co‐residence with a spouse/partner in LTC (OR = 0.334, 95% CI = 0.139–0.801, *p* = 0.014), healthier lifestyle (OR = 0.949, 95% CI = 0.925–0.973, *p <* 0.001) and better ADLs (OR = 0.972, 95% CI = 0.948–0.996, *p* = 0.025) were associated with lower odds of membership in this profile. By contrast, higher psychological resilience (OR = 1.028, 95% CI = 1.005–1.053, *p* = 0.017) was associated with greater odds of belonging to this profile.
3. Profile 3 (Moderate dementia fear—high concerns about family burden): The absence of co‐residence with a spouse/partner in LTC (OR = 0.284, 95% CI = 0.112–0.724, *p* = 0.008), absence of dementia family history (OR = 0.027, 95% CI = 0.009–0.082, *p <* 0.001), healthier lifestyle (OR = 0.962, 95% CI = 0.936–0.989, *p* = 0.006) and better ADLs (OR = 0.969, 95% CI = 0.944–0.995, *p* = 0.018) were associated with lower odds of membership in this profile.
4. Profile 4 (High dementia fear—moderate concerns about family burden): The absence of co‐residence with a spouse/partner in LTC (OR = 0.206, 95% CI = 0.071–0.598, *p* = 0.004), absence of dementia family history (OR = 0.024, 95% CI = 0.006–0.091, *p <* 0.001), healthier lifestyle (OR = 0.845, 95% CI = 0.809–0.883, *p <* 0.001) and better ADLs (OR = 0.956, 95% CI = 0.929–0.984, *p* = 0.002) were associated with lower odds of membership in this profile. By contrast, higher psychological resilience (OR = 1.096, 95% CI = 1.050–1.144, *p <* 0.001) was associated with greater odds of belonging to this profile.

**TABLE 4:** Multinomial logistic regression analysis of factors influencing the potential profiles of FODS.

Class	Variables	*β*	SE	Wald *χ* ^2^	*p*	OR	95% CI (LCL–UCL)
Profile 2	Sex						
	Male	−0.550	0.314	3.065	0.080	0.577	0.312–1.068
	Female^a^	—	—	—	—	—	—
	Living burden						
	Have a certain burden	−0.612	0.916	0.446	0.504	0.542	0.090–3.266
	Basically no burden	0.273	0.351	0.603	0.438	1.314	0.660–2.616
	No burden at all^a^	—	—	—	—	—	—
	Co‐residing with a spouse/partner in LTC						
	No (separated, single, divorced, widowed)	−1.098	0.447	6.025	0.014*	0.334	0.139–0.801
	Yes^a^	—	—	—	—	—	—
	Duration of stay in a nursing home						
	≤ 6 months	0.258	0.347	0.554	0.457	1.295	0.656–2.558
	6–12 months	1.247	0.662	3.545	0.060	3.481	0.950–12.749
	≥ 12 months^a^	—	—	—	—	—	—
	Family history of dementia						
	No	0.808	0.748	1.166	0.280	2.243	0.517–9.724
	Yes^a^	—	—	—	—	—	—
	DKAS	0.020	0.024	0.704	0.401	1.021	0.973–1.071
	CD‐RISC	0.028	0.012	5.655	0.017*	1.028	1.005–1.053
	HPLP‐IIR	−0.053	0.013	16.739	< 0.001***	0.949	0.925–0.973
	BI	−0.028	0.013	5.053	0.025*	0.972	0.948–0.996
Profile 3	Sex						
	Male	−0.112	0.343	0.107	0.744	0.894	0.456–1.752
	Female^a^	—	—	—	—	—	—
	Living burden						
	Have a certain burden	0.106	0.888	0.014	0.905	1.112	0.195–6.342
	Basically no burden	0.192	0.383	0.251	0.617	1.211	0.572–2.564
	No burden at all^a^	—	—	—	—	—	—
	Co‐residing with a spouse/partner in LTC						
	No (separated, single, divorced, widowed)	−1.258	0.477	6.951	0.008**	0.284	0.112–0.724
	Yes^a^	—	—	—	—	—	—
	Duration of stay in a nursing home						
	≤ 6 months	0.221	0.377	0.345	0.557	1.247	0.596–2.610
	6–12 months	0.182	0.775	0.055	0.814	1.200	0.263–5.482
	≥ 12 months^a^	—	—	—	—	—	—
	Family history of dementia						
	No	−3.626	0.576	39.633	< 0.001***	0.027	0.009–0.082
	Yes^a^	—	—	—	—	—	—
	DKAS	−0.003	0.027	0.012	0.913	0.997	0.945–1.052
	CD‐RISC	−0.007	0.013	0.319	0.572	0.993	0.968–1.018
	HPLP‐IIR	−0.039	0.014	7.641	0.006**	0.962	0.936–0.989
	BI	−0.032	0.013	5.611	0.018*	0.969	0.944–0.995
Profile 4	Sex						
	Male	−0.034	0.427	0.006	0.937	0.967	0.418–2.233
	Female^a^	—	—	—	—	—	—
	Living burden						
	Have a certain burden	0.204	1.063	0.037	0.848	1.226	0.153–9.841
	Basically no burden	0.328	0.462	0.504	0.478	1.389	0.561–3.436
	No burden at all^a^	—	—	—	—	—	—
	Co‐residing with a spouse/partner in LTC						
	No (separated, single, divorced, widowed)	−1.578	0.543	8.442	0.004**	0.206	0.071–0.598
	Yes^a^	—	—	—	—	—	—
	Duration of stay in a nursing home						
	≤ 6 months	0.348	0.466	0.558	0.455	1.417	0.568–3.531
	6–12 months	0.448	0.919	0.237	0.626	1.565	0.258–9.473
	≥ 12 months^a^	—	—	—	—	—	—
	Family history of dementia						
	No	−3.730	0.678	30.294	< 0.001***	0.024	0.006–0.091
	Yes^a^	—	—	—	—	—	—
	DKAS	0.059	0.041	2.051	0.152	1.060	0.979–1.149
	CD‐RISC	0.092	0.022	17.763	< 0.001***	1.096	1.050–1.144
	HPLP‐IIR	−0.168	0.022	57.101	< 0.001***	0.845	0.809–0.883
	BI	−0.045	0.015	9.454	0.002**	0.956	0.929–0.984

*Note:* Profile 1 = Low dementia fear—little concerns about family burden, Profile 2 = Low dementia fear—moderate concerns about family burden, Profile 3 = Moderate dementia fear—High concerns about family burden, Profile 4 = High dementia fear—moderate concerns about family burden; Profile 1 served as the reference category in multinomial logistic regression.

Abbreviations: BI, The Barthel index; CD‐RISC, Connor–Davidson Resilience Scale; DKAS, Dementia Knowledge Assessment Scale; HPLP‐IIR, The Health—Promoting Lifestyle Profile II; OR, odds ratio.

^a^ Reference category.

* *p* < 0.05.

** *p* < 0.01.

*** *p* < 0.001.

The complete statistical outputs are shown in Table 4. Some categories contained relatively few participants, which may have reduced the precision and stability of the corresponding estimates. Specifically, the 6‐ to 12‐month duration category included 45 participants, while only four participants in each of Profiles 1 and 2 reported a family history of dementia. The corresponding odds ratios and confidence intervals should therefore be interpreted cautiously because of limited precision and potential sparse‐data bias.

## 6 Discussion

This study employed LPA to identify heterogeneous profiles of dementia fear among older adults with multimorbidity residing in LTC facilities. A four‐profile solution demonstrated the best fit to the data. Specifically, the profiles were as follows: Profile 3, Moderate dementia fear—high concerns about family burden (40.0%), Profile 2, Low dementia fear—moderate concerns about family burden (33.2%), Profile 4, High dementia fear—moderate concerns about family burden (13.5%) and Profile 1, Low dementia fear—little concerns about family burden (13.3%). In this study, the ‘concerns about family burden’ used to characterise these profiles refers to the corresponding dimension of the Fear of Dementia Scale (FODS), which encompasses four directly measured domains: worries about caregiving burden, anxiety about inability to care for family members, concerns about institutional placement and financial burden. To our knowledge, this study is the first to identify distinct latent profiles of dementia fear in this specific population, multimorbid older adults. Further analysis demonstrated that profile membership was significantly associated with key factors that shape how multimorbid older adults experience and cope with dementia fear, including psychological resilience, health‐promoting behaviours, ADLs, co‐residing with a spouse/partner in LTC and family history of dementia.

Notably, our four‐profile model, developed exclusively for multimorbid older adults in LTC facilities, identified a distinct and novel latent profile: Profile 3, Moderate dementia fear—high concerns about family burden. This profile represents an unreported pattern in prior literature, marked by concurrent moderate dementia fear and disproportionately elevated concerns about family burden. Critically, this non‐linear pattern diverges from the simple linear association consistently reported by community‐based studies that have treated dementia fear as a unidimensional construct (Meyer et al. 2024; Wu et al. 2025). This discrepancy arises not only because single‐item and brief rating approaches, which are common in large population surveys, cannot capture the multidimensional structure of dementia fear (Chen et al. 2026), but also because the unique context of multimorbidity and institutional care reshapes how older adults weigh different dimensions of dementia fear, underscoring the unique value of LPA in elucidating such complex heterogeneity in this understudied population.

Beyond methodological insights, Profile 3 stands out as the most distinct group in our four‐profile solution among multimorbid older adults in LTC facilities, defined by moderate dementia fear paired with the highest level of concerns about family burden across all participants. Its unique manifestation can be attributed to the interplay of psychosocial factors and economic realities, which are already heightened by the ongoing experience of managing multiple chronic conditions, and further amplified by the institutional care context. What differentiates this profile most is that participants' concerns about family burden outweigh their fear of the disease itself, a pattern not observed in the other three groups. From a psychosocial perspective, the profound concerns about family burden in this group likely stem from a threat to the relational self and the anticipated loss of key familial roles (Mentzou et al. 2023). When one's self‐concept is deeply interwoven with the capacity to contribute to and care for the family, the prospect of dementia is feared not merely for its cognitive decline, but as a force that would sever these defining ties and reverse one's role from a contributor to a dependent, thereby stripping away one's ability to provide care (Toki et al. 2023). This threat to relational identity and agency can render fears about both becoming a family burden and losing the capacity to provide care more salient than dementia fear itself. Economically, catastrophic cognitions about dementia‐related care costs further intensify the financial burden sub‐domain of our measured concerns about family burden. These concerns are amplified by the ongoing financial strain of chronic disease management for older adults with multimorbidity. This perception is grounded in a stark reality: informal caregiving is projected to account for 60%–80% of the total costs of Alzheimer's disease and related dementias in China (Liu et al. 2025). Importantly, the overrepresentation of rural older adults in our sample may amplify these concerns, as institutional care costs in rural contexts often deplete their children's savings or grandchildren's education funds (Li et al. 2022). These real‐world perceptions directly explain the disproportionately elevated concerns about family burden that make Profile 3 unique in this multimorbid LTC population.

In this study, among older adults with multimorbidity in LTC facilities, participants not co‐residing with a spouse or partner had a significantly lower likelihood of membership in Profiles 2, 3 and 4. This finding differs from previous community‐based research suggesting that living with a spouse may be associated with lower dementia worry (Du et al. 2025). The discrepancy may reflect differences in care context. In community settings, spousal co‐residence may provide emotional and instrumental support. In LTC facilities, however, living with a spouse may increase daily exposure to the partner's declining health and heighten concerns about reciprocal caregiving, future dependency and financial or emotional burden. Thus, the role of spousal co‐residence may be context‐dependent rather than uniformly protective. For older adults with multimorbidity co‐residing with a spouse in LTC facilities, the bilateral care expectation may be particularly salient. They may not only depend on their spouse as a source of support but also feel obligated to care for their spouse in return, extending their worry from personal risk to the dual care pressure of potential illness in both partners (Shaw and Csikai 2024). This may further exacerbate dementia fear and concerns about family care burden. Residents co‐residing with a spouse or partner may warrant particular attention in clinical assessment, and couple‐focused psychological support may be considered as a potential approach to alleviating their concerns about spousal care burden.

In this study, among older adults with multimorbidity in LTC facilities, participants with a family history of dementia had a significantly higher likelihood of membership in Profiles 3 and 4, whereas no significant association was observed with Profile 2. Consistent with previous studies among community‐dwelling older adults, a family history of dementia was associated with greater dementia fear (Chen et al. 2026). Our findings extend this evidence by suggesting that this association was not uniform across all participants but was concentrated in profiles characterised by elevated fear. This profile‐specific pattern indicates that family history may be particularly useful as an initial screening marker for identifying residents who require further psychological assessment. Nevertheless, this finding should be interpreted cautiously because of the small number of participants reporting a family history in some profiles.

Two potential mechanisms may account for these observations. First, dementia has well‐established genetic susceptibility. Those with a family history of dementia may be more likely to harbour concerns about carrying relevant pathogenic genes, which contributes to their perceived elevated risk of developing the disease in the future (Coley et al. 2021). For older adults with multimorbidity in LTC facilities, this uncertainty regarding potential threats to one's own health may trigger intense inner fear. Second, first‐hand, long‐term exposure to the progressive cognitive and functional deterioration of relatives with dementia may foster a more tangible and enduring cognitive schema regarding one's own dementia susceptibility, thereby intensifying dementia fear (Heser et al. 2023). No significant association was observed between family history of dementia and membership in Profile 2. Profile 2 is characterised by low levels of dementia fear and moderate concerns about family burden, suggesting that participants in this group may have a lower level of perceived risk of developing dementia themselves. Therefore, even in the presence of a family history, it may be difficult for this factor to exert a significant effect in the absence of the core prerequisite of elevated dementia fear. This finding further supports the clinical heterogeneity across the latent profiles identified in this study. For residents in Profiles 3 and 4, family history‐related dementia fears may warrant particular attention in clinical assessment and support. For Profile 2, which is characterised by low dementia fear but moderate concerns about family burden, the focus may be on alleviating participants' concerns about family burden.

Psychological resilience was associated with dementia fear profile membership in a non‐linear and profile‐specific manner. Previous studies have generally reported an inverse association between psychological resilience and dementia fear, suggesting that resilience may serve as a protective psychological resource (Dai et al. 2023). Our findings provide a more nuanced perspective. Compared with Profile 1, greater resilience was associated with higher adjusted odds of membership in Profiles 2 and 4, but not Profile 3. However, these adjusted associations should be interpreted with caution, as the unadjusted mean CD‐RISC scores did not differ significantly between Profile 1 and Profile 2 or between Profile 1 and Profile 4 in the post hoc comparisons. The bivariate correlation between CD‐RISC and HPLP‐IIR was weak (*r* = 0.092), and multicollinearity diagnostics were acceptable (all VIF values < 1.601), indicating that collinearity was unlikely to account for this discrepancy. Rather, the discrepancy is likely to reflect the fact that the unadjusted comparisons and the adjusted models address different questions, and that adjustment for variables strongly related to profile membership, particularly health‐promoting lifestyle, altered the estimate for resilience. The association therefore emerged only after adjustment and warrants cautious interpretation. Against this background, the descriptive pattern across profiles remains informative. Profiles 2 and 4 both exhibited moderate concerns about burdening family members. These concerns exceeded those reported in Profile 1, despite Profiles 2 and 4 occupying opposite ends of the overall dementia fear continuum. Together with the null finding for Profile 3, this indicates that psychological resilience was not linearly aligned with the overall severity of dementia fear. Instead, its association differed across distinct configurations of fear dimensions.

One possible explanation is that resilience reflects the capacity to adapt to and engage with adversity rather than the absence of concern (Southwick et al. 2014). Older adults with greater resilience may remain aware of the possible consequences of cognitive decline and may be more willing to acknowledge concerns about future dependency and its potential impact on their families. From this perspective, moderate concerns about burdening family members observed in Profiles 2 and 4 may represent a realistic appraisal of anticipated care needs rather than psychological vulnerability alone. However, cognitive appraisal, coping strategies and willingness to disclose concerns were not directly measured in this study, so this interpretation remains tentative. Alternatively, the pattern may be partly statistical, reflecting the joint contribution of resilience and other variables that distinguish the profiles rather than an independent effect of resilience alone.

The absence of a significant association between resilience and Profile 3 should also be interpreted cautiously. This finding does not confirm that resilience functions differently within Profile 3, because the study did not formally test whether regression coefficients differed across profiles. Profile 3 had the lowest mean CD‐RISC score of the four profiles. Despite this, the null finding emerged only after adjustment for factors that most clearly distinguished Profile 3 from the other profiles, particularly family history of dementia and co‐residence with a spouse or partner, which appear to account for the unadjusted difference. Longitudinal studies incorporating measures of coping, threat appraisal and family‐related concerns are needed to clarify how resilience relates to the development and expression of different dimensions of dementia fear. These findings suggest that greater resilience should not be assumed to indicate an absence of dementia fear, and that resilience and specific fear dimensions may need to be considered together in assessment. Given the profile‐specific pattern observed across the latent profiles and the instability of the resilience estimates between the unadjusted and adjusted analyses, this association should be considered exploratory.

Among older adults with multimorbidity in LTC facilities, participants who adopted a healthier lifestyle had a significantly lower likelihood of membership in Profiles 2, 3 and 4. This association is broadly consistent with previous research linking health‐promoting behaviours to lower dementia worry (Safiri et al. 2024), providing confirmatory evidence in a previously understudied population, namely institutionalised older adults with multimorbidity. In addition, our findings extend previous variable‐centred research by showing that a healthy lifestyle was associated not only with lower overall fear levels but specifically with membership in profiles characterised by lower dementia fear and family burden concerns. A healthy lifestyle embodies an active coping stance that may help individuals gain a sense of disease controllability through health self‐management (Hirooka et al. 2021), leading them to recognise that their health can be modified through actions and thereby mitigating dementia fear (Safiri et al. 2024). These findings suggest that promoting healthy lifestyles in LTC facilities, such as establishing health activity groups and personalised plans tailored to residents' chronic condition status and functional capacity, may be a useful strategy for supporting psychological well‐being related to dementia risk. In particular, strengthening awareness of active health management among high‐risk groups across all dementia fear profiles may help maximise its beneficial association with psychological well‐being.

Among older adults with multimorbidity in LTC facilities, participants with poorer functioning in ADLs had a significantly higher likelihood of membership in Profiles 2, 3 and 4. This association is broadly consistent with previous research linking functional impairment and reduced independence to greater dementia worry (Meyer et al. 2024), providing confirmatory evidence in institutionalised older adults with multimorbidity. In addition, our findings extend previous variable‐centred research by showing that poorer ADLs were associated specifically with membership in profiles characterised by more prominent dementia fear and family burden concerns, rather than simply with higher overall fear scores. A decline in daily living abilities, often the cumulative outcome of progressive organ and functional damage caused by multiple chronic conditions, may increase individuals' subjective perceptions of dementia risk and future care dependence. Functional impairment may be associated with a greater perceived need for future family care, a need that is already elevated by the day‐to‐day support requirements of managing multiple chronic conditions, and may be further amplified by the prospect of irreversible dementia‐related dependence (Choi et al. 2024). A decline in ADLs may also be perceived as an intuitive precursor to cognitive decline, which is associated with stronger perceptions of the severity and irreversibility of dementia (Chae 2024) and higher levels of dementia fear. These findings suggest that providing customised functional rehabilitation training tailored to residents' chronic condition status and functional capacity, along with information about respite services for potential family caregivers, may help address both perceived care dependence and dementia fear. Such support may be particularly relevant for residents in profiles with more prominent dementia fear and family burden concerns.

### 6.1 Implications for Nursing Practice

The findings highlight the heterogeneity of dementia fear among older adults with multimorbidity in LTC facilities, suggesting that a uniform approach to psychological support may not adequately address residents' differing needs. Nurses may incorporate dementia fear assessment into routine care, attending to both overall fear levels and specific concerns about becoming a burden on family members. Factors associated with profile membership, including family history of dementia, spousal co‐residence, health‐promoting lifestyles and functional status, may help identify residents needing further assessment, though they should not be interpreted as causal predictors. Psychological resilience showed a complex, profile‐specific association that emerged only after adjustment, and its role in routine assessment requires further investigation. Residents in the moderate dementia fear–high family burden concerns profile, the largest subgroup (40.0%), may need targeted support for future dependency and burden concerns, including health‐promoting lifestyle support, maintenance of functional independence and anticipatory care planning. Those in the high dementia fear–moderate family burden concerns profile may benefit from more intensive assessment and individually tailored psychological support, especially with a family history of dementia. For residents with low overall fear but moderate burden concerns, nurses may focus on exploring the sources of concerns and providing information about available care and family support resources. At the organisational level, this profile‐based approach supports more targeted and efficient care delivery. It can inform routine psychological screening pathways and care resource allocation in LTC facilities and guide nurse training to improve recognition of heterogeneous dementia fear presentations and person‐centred communication skills, ultimately delivering more precise stratified support for residents. As the present study did not evaluate any interventions, the effectiveness of these profile‐informed approaches should be examined in future longitudinal and intervention studies.

### 6.2 Strengths and Limitations

A major strength of this study is the application of a person‐centred LPA approach. This approach enabled the identification of distinct dementia fear profiles among older adults with multimorbidity in LTC facilities. The findings may provide useful insights for developing more targeted, profile‐informed nursing interventions aimed at supporting psychological well‐being among this vulnerable population. Despite this strength, several limitations should be acknowledged. First, the research was limited to 25 LTC facilities across 10 districts/counties in Tangshan, Hebei Province, China. This geographic confinement, combined with the exclusive focus on institutionalised older adults, may restrict the generalisability of findings to other regions, community‐dwelling older adults with multimorbidity or populations in socioculturally distinct contexts. Meanwhile, this study employed cluster sampling across 25 LTC facilities, creating a nested data structure. The dataset did not include facility‐level identifiers, which precluded the application of cluster‐robust standard errors or multilevel modelling. Standardised data collection procedures were implemented across all facilities to ensure consistent data collection, but these procedures cannot account for within‐facility correlation. Consequently, the standard errors may be underestimated, and the statistical significance of some associations, particularly those close to the conventional threshold, should be interpreted cautiously. Future studies should record facility identifiers and employ multilevel or cluster‐robust analytic methods. Second, although key demographic and health behaviour variables were included, specific details of multimorbidity such as chronic disease types, severity and symptom burden were not accounted for. These omissions may obscure associations between comorbidity profiles and dementia fear subtypes. Third, despite an adequate total sample size (*n* = 557) for LPA, some identified classes had small proportions (e.g., 13.3% and 13.5%), potentially limiting the subgroup analyses' statistical power. Future studies require larger samples with proportional class distribution to validate profile stability. Fourth, the proportion of participants who had questionnaire items read aloud by researchers was not recorded. Because dementia fear and shame items may be answered differently when read aloud by a researcher rather than self‐completed, this may have introduced variability in responses that could not be quantified. Finally, the use of an unadjusted MMSE cut‐off (< 24) without education stratification may have misclassified some cognitively intact older adults with low educational attainment, potentially affecting sample composition and generalisability.

## 7 Conclusion

This study identified four distinct latent profiles of dementia fear among older adults with multimorbidity in LTC facilities: low dementia fear—little concerns about family burden (13.3%), low dementia fear—moderate concerns about family burden (33.2%), moderate dementia fear—high concerns about family burden (40.0%), and high dementia fear—moderate concerns about family burden (13.5%). Profile membership was significantly associated with family history of dementia, co‐residing with a spouse/partner in LTC, psychological resilience, healthy lifestyle and ADLs. Findings from this classification and correlation analysis support the identification of high‐risk subgroups (e.g., the high dementia fear—moderate concerns about family burden group) and the factors linked to each profile. These findings may provide a basis for identifying high‐risk subgroups and informing the development of profile‐specific nursing interventions. Given the cross‐sectional design, the observed associations should not be interpreted as causal; whether profile‐specific interventions can alleviate dementia fear and improve quality of life in this population remains to be examined in future longitudinal and intervention studies.

## Author Contributions

**Guiyun Wang:** resources, writing – original draft, conceptualization, supervision, project administration. **Yuehua Han:** writing – original draft, conceptualization, formal analysis, software, visualization. **Qirong Chen:** writing – original draft, resources, writing – review and editing, supervision, methodology. **Yanxia Qi:** formal analysis, software. **Jinke Kuang:** data curation, investigation. **Chaowei Guo:** data curation, investigation. **Shuxing Li:** writing – original draft, writing – review and editing, funding acquisition, validation.

## Funding

The authors have nothing to report.

## Ethics Statement

This study was conducted in accordance with the Declaration of Helsinki. Ethical approval was granted by the Ethics Review Committee of North China University of Science and Technology (approval number: 20211104, approval date: 4 November 2021). Participants were fully informed of the study objectives, the voluntary nature of participation, and the protocols implemented to protect confidentiality and anonymity. All questionnaires were assigned unique numerical identifiers with no personally identifiable information, and no identifiable personal data will appear in any published outputs.

## Consent

Written informed consent was obtained from all participants after they fully understood the purpose and procedures of the study.

## Conflicts of Interest

The authors declare no conflicts of interest.

## Supporting information


**Figure S1:** Line plot of LPA fit indices for the FODS.

## Data Availability

The datasets generated and analysed during the current study are available from the corresponding author on reasonable request.
