# Supplementary material for: Dementia Fear Profiles and Influencing Factors Among Older Adults With Multimorbidity in Long‐Term Care Facilities
Source: Nurs Open. 2026 Nov 7;13(11):e71023. doi: 10.1002/nop2.71023 (PMC13744008; doi:10.1002/nop2.71023)
Supplement: Supplementary file 1 [file NOP2-13-e71023-s001.docx]

**FIGURE S1** Line plot of LPA fit indices for the FODS.
